# Ascertainment and Verification of End-Stage Renal Disease and End-Stage Liver Disease in the North American AIDS Cohort Collaboration on Research and Design

**DOI:** 10.1155/2015/923194

**Published:** 2015-02-19

**Authors:** Mari M. Kitahata, Daniel R. Drozd, Heidi M. Crane, Stephen E. Van Rompaey, Keri N. Althoff, Stephen J. Gange, Marina B. Klein, Gregory M. Lucas, Alison G. Abraham, Vincent Lo Re, Justin McReynolds, William B. Lober, Adell Mendes, Sharada P. Modur, Yuezhou Jing, Elizabeth J. Morton, Margaret A. Griffith, Aimee M. Freeman, Richard D. Moore

**Affiliations:** ^1^Division of Allergy and Infectious Diseases, Department of Medicine, University of Washington, Seattle, WA 98109, USA; ^2^Center for AIDS Research, University of Washington, Seattle, WA 98104, USA; ^3^Bloomberg School of Public Health, Johns Hopkins University, Baltimore, MD 21205, USA; ^4^Division of Infectious Diseases Chronic Viral Illness Service, Department of Medicine, McGill University, Montreal, QC, Canada; ^5^Department of Medicine, Johns Hopkins University, Baltimore, MD 21287, USA; ^6^Center for Clinical Epidemiology and Biostatistics, University of Pennsylvania School of Medicine, Philadelphia VA Medical Center, Philadelphia, PA 19104, USA; ^7^Medical Education and Biomedical Informatics, School of Medicine, University of Washington, Seattle, WA 98109, USA; ^8^Biobehavioral Nursing and Health Systems, Department of Nursing, University of Washington, Seattle, WA 98195, USA

## Abstract

The burden of HIV disease has shifted from traditional AIDS-defining illnesses to serious non-AIDS-defining comorbid conditions. Research aimed at improving HIV-related comorbid disease outcomes requires well-defined, verified clinical endpoints. We developed methods to ascertain and verify end-stage renal disease (ESRD) and end-stage liver disease (ESLD) and validated screening algorithms within the largest HIV cohort collaboration in North America (NA-ACCORD). Individuals who screened positive among all participants in twelve cohorts enrolled between January 1996 and December 2009 underwent medical record review to verify incident ESRD or ESLD using standardized protocols. We randomly sampled 6% of contributing cohorts to determine the sensitivity, specificity, positive predictive value (PPV), and negative predictive value (NPV) of ESLD and ESRD screening algorithms in a validation subcohort. Among 43,433 patients screened for ESRD, 822 screened positive of which 620 met clinical criteria for ESRD. The algorithm had 100% sensitivity, 99% specificity, 82% PPV, and 100% NPV for ESRD. Among 41,463 patients screened for ESLD, 2,024 screened positive of which 645 met diagnostic criteria for ESLD. The algorithm had 100% sensitivity, 95% specificity, 27% PPV, and 100% NPV for ESLD. Our methods proved robust for ascertainment of ESRD and ESLD in persons infected with HIV.

## 1. Introduction

Antiretroviral therapy (ART) has transformed HIV infection from a rapidly progressive fatal illness to a manageable chronic disease [1]. However, mortality may remain elevated compared to HIV-negative individuals [2–4] as HIV-infected individuals confront an increasing burden of comorbid conditions commonly seen in the aging general population including malignancies and cardiovascular, renal, and liver diseases [5–14]. Federal US HIV/AIDS policy has prioritized the study of these age-related conditions in persons infected with HIV [15, 16], yet research on HIV-related comorbid disease has been limited by inconsistent diagnostic criteria, reliance on administrative diagnosis data, and lack of verified, definitive clinical outcomes [10–14, 17–31].

Renal disease is common in HIV-infected individuals and spans a spectrum of severity of illness [32]. End-stage renal disease (ESRD), defined as irreversible kidney damage treated with renal replacement therapy (RRT), represents the most significant and definitive clinical endpoint. Many known risk factors for ESRD including diabetes mellitus [33], hypertension [34], and hepatitis C virus (HCV) coinfection [35] are more common in HIV-infected individuals. There are no definitive criteria for ascertainment or verification of ESRD in persons with HIV infection. Inferences from previous studies of ESRD have been limited by the use of incomplete laboratory data [10, 11], composite endpoints [11, 12, 29, 31], and focus on a single center [31] or clinical trial setting [20].

End-stage liver disease (ESLD) is the final and often terminal result of chronic liver disease. ESLD-related deaths have increased as a percentage of total deaths amongst HIV-infected individuals [21]. Prevalence of hepatitis B virus (HBV) [36–38] and hepatitis C virus (HCV) coinfection [39, 40] and alcohol abuse [41, 42], all leading causes of ESLD, are increased in persons infected with HIV. ART reduces progression to liver fibrosis in individuals coinfected with HCV [43, 44] and the advent of highly effective direct acting agents (DAAs) marks the beginning of a new HCV treatment era. However, research aimed at improving liver disease outcomes among HIV-infected individuals requires well-defined, clinical ESLD endpoints.

Previous studies of ESLD have used heterogeneous screening criteria and case definitions and focused on specific subpopulations [13, 14, 25, 26] or patients who have undergone liver biopsy [45], thereby introducing potential selection bias. Both the American Association for the Study of Liver Disease (AASLD) and the European Association for the Study of the Liver (EASL) have published guidelines that define diagnoses consistent with ESLD (ascites, spontaneous bacterial peritonitis (SBP), esophageal/gastric variceal hemorrhage, hepatic encephalopathy, and hepatocellular carcinoma), which rely on the presence of one or more clinical events, physical examination, and laboratory, radiographic, or endoscopic findings. Only one study has examined the utility of screening for ESLD among persons infected with HIV, which was conducted in the Veterans Aging Cohort [46].

The North American AIDS Cohort Collaboration on Research and Design (NA-ACCORD) developed standardized protocols to identify and verify four clinically important outcomes in HIV-infected individuals (e.g., myocardial infarction (MI) [47], malignancies, ESRD, and ESLD) and designed web-based applications to improve the efficiency of endpoint verification. In this study, we examined the accuracy and completeness of novel screening algorithms to identify ESRD and ESLD events using routinely collected clinical data in the large and diverse population of HIV-infected individuals in NA-ACCORD. We used a case-cohort design to rigorously test the discriminatory properties of screening protocols and report on the sensitivity, specificity, and negative predictive value (NPV) and positive predictive value (PPV) of algorithms for identifying ESRD and ESLD events validated through comprehensive medical record review using standardized criteria.

## 2. Materials and Methods

### 2.1. Study Population

NA-ACCORD is a consortium of HIV cohorts from North America and one of seven regional collaborations of the International Epidemiologic Databases to Evaluate AIDS (IeDEA) supported by the National Institutes of Health. Details on this collaboration have been published previously [48]. Briefly, NA-ACCORD is the largest and most diverse cohort of persons infected with HIV in North America and consists of 25 cohorts that collect data on >130,000 HIV-infected individuals from more than 200 clinical sites in the US and Canada. These sites reflect the spectrum of HIV disease in North America and include health maintenance organizations, county hospitals, academic medical centers, and private practices in the US and Canada. Each cohort submits standardized clinical data at scheduled intervals including demographic characteristics, medications, laboratory values, and diagnoses on enrolled participants. All patients enrolled between January 1996 and December 2009 in twelve clinical cohorts were included in this study. Whereas previous studies have relied on administrative ICD-9-CM coded billing data, NA-ACCORD captures clinical diagnoses documented prospectively by the treating clinician in the medical record. These data are transferred securely to the NA-ACCORD Data Management Core (DMC) where they undergo quality control for completeness and accuracy and are combined into a harmonized relational database. The human subject activities of the NA-ACCORD and of each of the participating cohort studies have been reviewed and approved by their respective local institutional review boards.

### 2.2. Data Collection

The NA-ACCORD DMC developed web-based applications to standardize ESRD and ESLD event verification and data collection across cohorts. The web-based platform facilitates secure access to authorized data and reduces administrative time, thereby reducing costs. Medical record review was performed by or under the supervision of a physician at each cohort. Reviewers were presented with potential ESLD or ESRD cases identified in his/her cohort using the screening algorithms (described below) applied centrally by the NA-ACCORD Epidemiology/Biostatistics Core. Diagnoses, medications, procedures, laboratory test results, and other relevant clinical data for each potential case were prepopulated into the application to increase the efficiency of review. The reviewer answered structured questions to verify or invalidate the potential case using drop-down menus, radio-buttons, and checkboxes to ensure the integrity of the data. Electronic data entry facilitated automated checks for missing data.

### 2.3. Outcome Screening and Verification Procedures

Screening and verification criteria were developed by NA-ACCORD ESRD and ESLD Working Groups comprising individuals with clinical and epidemiologic expertise in these areas. Comprehensive review of all available medical records was conducted for each individual who screened positive for ESRD or ESLD to confirm the event using a standardized protocol. In those with no evidence of ESRD or ESLD, the absence of the condition was explicitly recorded.

#### 2.3.1. ESRD Screening Criteria

We identified potential ESRD cases using either diagnosis or laboratory criteria consistent with ESRD in HIV-infected individuals [49] outlined below.

- *Diagnosis Criteria*. Diagnosis criteria include any single clinician-documented diagnostic or procedure code consistent with ESRD (see Appendix).
- *Laboratory Criteria*. Positive laboratory screening criteria included at least two estimated glomerular filtration rate (eGFR) measurements of <30/mL/min/1.73 m^2^ separated by greater than 90 days without an intervening measure ≥30 mL/min/1.73 m^2^. eGFR was calculated using the Chronic Kidney Disease Epidemiology Collaboration (CKD-EPI) equation which incorporates age, race, and sex [50].

#### 2.3.2. ESRD Verification

Criteria used to confirm ESRD are shown in Table 1. Each potential case identified by diagnosis, procedure, or laboratory criteria listed above underwent validation for ESRD by review of all available medical records using a standardized protocol to confirm evidence of RRT defined as chronic dialysis (hemo- or peritoneal dialysis) of greater than six months duration, arteriovenous fistula (AVF) placement with evidence of dialysis, or renal transplantation. Dialysis delivered temporarily (less than six months) for acute kidney injury in hospitalized individuals was not considered ESRD. Dates of renal transplantation, dialysis initiation or AVF placement, confirmation source, kidney biopsy reports, medication use, and substance use were all abstracted from medical records as part of the validation process and recorded in the centralized web-based data entry application. ESRD date was defined as the earliest confirmed date of RRT.

#### 2.3.3. ESLD Screening Criteria

Criteria for ESLD ascertainment included two noninvasive laboratory-based measures of hepatic fibrosis that have been validated in HIV-infected individuals (aspartate aminotransferase (AST)/platelet ratio index (APRI) [51] and FIB-4 [52]) but have not previously been examined for use as screening criteria in this population. The APRI is comprised of the AST and platelet count, and the FIB-4 combines age, platelet count, alanine aminotransferase (ALT), and AST to create an index. Both measures identify advanced hepatic fibrosis/cirrhosis. Applying cut-offs used in previous studies, a positive screen was defined as having at least two APRI scores >1.5 or two FIB-4 scores >3.25, greater than 6 months apart. A positive laboratory screen for impaired hepatic function required two of the following laboratory values: total bilirubin ≥0.28 mmol/L (≥5.0 mL/dL), albumin <0.11 mmol/L (<2.0 mg/dL), or INR >1.7, greater than 6 months apart. Those who screened positive for ESLD met either diagnosis or laboratory-based criteria outlined below.

- * Diagnosis Criteria*. Diagnosis criteria include any single clinician-documented diagnostic or procedure code consistent with ESLD (see Appendix).
- *Laboratory and Fibrosis Criteria*. In order to meet the laboratory criteria to screen positive, a patient needed to have both
  - a positive lab-based index for advanced hepatic fibrosis (APRI or FIB-4);
  - at least one other laboratory abnormality consistent with impaired hepatic function (e.g., total bilirubin, albumin, and INR).

#### 2.3.4. ESLD Verification

Each potential case identified by diagnosis and laboratory criteria above underwent validation for ESLD by review of all available medical records using a standardized protocol to confirm evidence of one the following diagnoses: ascites, SBP, variceal hemorrhage, hepatic encephalopathy, or hepatocellular carcinoma. Confirmation of one of these diagnoses met criteria for verified ESLD based on AASLD and EASL ESLD case definitions (Table 1) and the ESLD date was defined as the earliest confirmed diagnosis date. Confirmation source (e.g., radiographic or endoscopic reports), liver biopsy reports, medication use, and substance use were all abstracted from medical records as part of the validation process and recorded in the centralized web-based data entry application.

### 2.4. Randomly Selected Subcohort

We performed comprehensive medical record review on a randomly selected sample of 9% of participants from contributing cohorts, termed the “subcohort” [53], to validate screening algorithms for ESRD and ESLD. Given the large number of participants in NA-ACCORD, medical record review of the entire cohort collaboration was not feasible. Two participating cohorts were unable to complete medical record review and one cohort with a large sample size was only able to complete medical record review for one-half of its selected sample. The final subcohort used to validate ESRD and ESLD screening criteria included 2,415 (6%) and 2,422 (6%) participants, respectively.

### 2.5. Data Analysis

We computed sensitivity, specificity, PPV, and NPV of the screening algorithm for ESRD and ESLD in the subcohort participants. Sensitivity was calculated as the proportion of individuals with verified events who screened positive. Specificity was calculated as the proportion of individuals without verified events who screened negative. PPV was calculated as the proportion of screened-positive individuals with a validated event. NPV was calculated as the proportion of screened-negative individuals without a validated event. For ESRD and ESLD, we conducted sensitivity analysis of the screening criteria by separating the criteria as (a) a diagnosis code or a laboratory value; (b) a diagnosis code with or without a laboratory value; (c) a laboratory value with or without a diagnosis code; or (d) both a diagnosis code and a laboratory value. For ESLD, two additional sensitivity analyses were conducted: (a) limiting the diagnosis criteria to the 3 most commonly used codes (ascites, SBP, or variceal hemorrhage) and (b) determining the utility of including procedure codes (liver transplant, paracentesis, and transjugular intrahepatic portosystemic shunt (TIPS)) in our screening algorithm.

## 3. Results

### 3.1. Participants' Characteristics

Demographic characteristics of individuals who underwent screening for ESRD, ESLD, and the subcohort are shown in Table 2. The proportion of non-Hispanic black individuals was higher among those who screened positive and had confirmed ESRD compared to those who screened negative. The proportion of individuals coinfected with HBV or HCV was higher among those who screened positive and had confirmed ESLD compared to those who screened negative.

### 3.2. End-Stage Renal Disease

A total of 43,433 patients from 12 cohorts contributed to the ESRD validation study of which 822 screened positive for ESRD by either diagnosis or laboratory criteria and underwent comprehensive medical record review. Two hundred and eighteen individuals were identified by diagnosis criteria alone, 622 were identified by laboratory criteria alone, and 18 were identified by both diagnosis and laboratory criteria. Of the 822 individuals who screened positive overall, 620 met clinical criteria for ESRD. Of the 620 verified cases of ESRD, 159 screened positive by diagnosis criteria, 473 by laboratory criteria, and 12 by both diagnosis and laboratory criteria.

None of the individuals who screened negative for ESRD in the randomly selected subcohort (*n* = 2,339) had verified ESRD. Overall, screening by either diagnosis or laboratory criteria had 100% sensitivity, 99% specificity, 82% PPV, and 100% NPV. Examined separately, diagnosis criteria were much less sensitive (27%) than laboratory criteria (77%), but the specificity and PPV for each was similar to the combined criteria as shown in Table 3. Requiring that both diagnosis and laboratory criteria be met to be classified as screened positive substantially improved the PPV to 100% though sensitivity was substantially diminished (5%).

### 3.3. End-Stage Liver Disease

A total of 41,463 patients from 12 cohorts contributed to the ESLD validation study of which 2,024 screened positive by either diagnosis or laboratory criteria and underwent comprehensive medical record review. Of these, 1,784 individuals were identified by diagnosis criteria alone, 447 by laboratory criteria alone, and 207 by both diagnosis and laboratory criteria. Of the 2,024 individuals who screened positive overall, 645 met diagnostic criteria for ESLD. Of the 645 verified cases identified by either diagnosis or laboratory criteria, 610 were identified by diagnosis criteria alone, 136 by laboratory criteria alone, and 101 by both diagnosis and laboratory criteria.

None of the 2,268 individuals who screened negative for ESLD in the subcohort had verified ESLD. Overall, screening by either diagnosis or laboratory criteria had 100% sensitivity, 95% specificity, 27% PPV, and 100% NPV as shown in Table 3. Examined separately, diagnosis criteria were highly sensitive (95%) and specific (96%), while laboratory criteria were less sensitive (20%) but highly specific (99%), and PPV for each were similar (29% and 22%, resp.). Requiring that both diagnosis and laboratory criteria be met to be classified as screened positive substantially improved the specificity and PPV to 100% and 35%, respectively, but decreased the sensitivity (15%).

In sensitivity analyses, 385 (63%) of the 610 ESLD events identified by diagnosis criteria were identified by a restricted set of diagnoses that included ascites, SBP, or esophageal varices resulting in greater specificity (98%) and PPV (39%), but lower sensitivity (59%). The addition of procedure codes (liver transplant, paracentesis, and TIPS) did not improve the sensitivity of ascertainment over diagnosis and laboratory criteria and, thus, was not included in the overall algorithm.

## 4. Discussion

We developed novel methods to identify and verify ESRD and ESLD that proved robust in the largest and most diverse cohort collaboration of persons infected with HIV in North America, thus being widely applicable to diverse cohorts of HIV-infected individuals to decrease misclassification and improve the validity of inferences from clinical research conducted in this population. Both ESRD and ESLD represent definitive clinical outcomes and add to the collection of adjudicated endpoints available for research within the NA-ACCORD.

The specificity and PPV of the screening algorithm for ESRD were higher than for ESLD, likely due to the specific nature of RRT and decreased creatinine clearance for ESRD, while the sensitivity of clinical diagnoses alone to identify ESRD was poor. Screening for ESLD relies on less specific markers of liver disease. The inclusion of the APRI and FIB-4 in our laboratory criteria is an important advance as the presence of advanced hepatic fibrosis and cirrhosis necessarily precedes the development of ESLD. To our knowledge, our study is the first to examine these measures for use in ascertainment of ESLD. Combining laboratory markers of advanced liver fibrosis with markers of impaired hepatic function maximized the specificity of ESLD ascertainment, but at the expense of sensitivity. As expected, limiting the diagnoses to ascites, SBP, or esophageal varices improved specificity but decreased sensitivity. Procedures that are specific for ESLD, such as liver transplantation and TIPS, were performed infrequently in clinical practice and, thus, did not add to the sensitivity of screening.

### 4.1. Strengths

Our study has several strengths. It was conducted in the largest, most diverse cohort of persons infected with HIV in the US and Canada making results generalizable across care settings and reflective of the burden of ESLD and ESRD among HIV-infected individuals in North America. Other key strengths include the completeness of inpatient and outpatient clinical data captured from the contributing cohorts, which decreases the likelihood of missing data; the use of standard procedures to harmonize clinical data across sites; systematic centralized ascertainment of potential cases; and standardized protocols for endpoint verification, which minimize misclassification. In addition, we conducted thorough medical record review of a large randomly selected subcohort of individuals to determine the sensitivity, specificity, PPV, and NPV of the screening algorithm for ESRD and ESLD. In order to provide the most rigorous estimates, all calculations were based on the conservative assumption that only those individuals who underwent medical record review were event-free. Comprehensive medical record review conducted for all participants in the randomly selected subcohort facilitates future case-cohort analyses conducted in NA-ACCORD.

### 4.2. Limitations

This study has several limitations. First, it is possible that we missed patients within the cohort with ESRD or ESLD. However, thorough review of medical records for over 2,400 cohort participants who were found to be event-free minimized this risk. Second, we may have misclassified confirmatory events as diagnostic of ESLD or ESRD when, in fact, they were due to other causes. We minimized the risk of misclassification by applying standardized criteria and structured data protocols to define each type of confirmatory events and referring ambiguous or questionable events to the DMC for review.

### 4.3. CNICS Cohort

We have extended the ESRD and ESLD ascertainment and verification protocols used in NA-ACCORD to multiple sites in the Centers for AIDS Research Network of Integrated Clinical Systems (CNICS) cohort [54] which includes >30,000 HIV-infected individuals in care from 1995 to the present at eight clinical sites across the US. Applying the same protocol and outcome definitions across NA-ACCORD and CNICS strengthens future collaborations using combined data for analyses. While there is some overlap between the 2 cohorts, each also has independent sites greatly enhancing the potential analytic power. In addition, data collected in the 2 cohorts are complementary. NA-ACCORD provides the very large sample size required to answer key questions related to HIV and ESRD and ESLD that cannot be addressed in smaller cohorts. CNICS provides detailed patient reported data such as the routine measurement of behavioral risk factors not available in other cohorts.

## 5. Conclusions

In conclusion, we developed algorithms to identify ESRD and ESLD using routinely collected clinical data and standardized protocols implemented via web-based applications to verify events in the largest and most diverse cohort of persons infected with HIV in North America. Methods developed in NA-ACCORD to identify and confirm ESRD and ESLD are broadly applicable to observational cohort studies and will facilitate research aimed at understanding the underlying mechanism and progression of the changing clinical spectrum of HIV disease.

## Figures and Tables

**Table 1:** Verification criteria for end-stage renal disease and end-stage liver disease.

Criteria for end-stage renal disease	

Hemodialysis/peritoneal dialysis	Provider documentation of chronic dialysis (>6 mos) in dialysis records, inpatient notes, outpatient clinic notes, or discharge summaries.

Kidney transplant	Provider documentation of kidney transplant in inpatient notes, outpatient clinic notes, or discharge summaries.

Criteria for end-stage liver disease	

Ascites	Abdominal ultrasound report indicating ascites
	Abdominal CT report indicating ascites
	Abdominal MRI report indicating ascites
	Abdominal peritoneal fluid analysis result from paracentesis
	Provider documentation of ascites identified by any procedure listed above without the corroborating primary radiology or laboratory report

Variceal hemorrhage	Esophagogastroduodenoscopy** (**EGD) report of active variceal bleeding
	EGD report of recent variceal bleeding
	EGD report of nonbleeding varices in the setting of acute gastrointestinal bleeding without other causes identified
	Provider documentation of variceal hemorrhage identified by EGD procedure without corroborating primary EGD report

Spontaneous bacterial peritonitis	Ascitic fluid culture with bacterial growth
	Ascitic fluid absolute neutrophil count ≥ 250 cells/mm^3^

Hepatic encephalopathy	Mental confusion consistent with hepatic encephalopathy documented in a progress note of a patient with known chronic liver disease *plus *absence of any of the following conditions:
	(i) intracranial lesions, such as subdural hematoma, intracranial bleeding, stroke, tumor, and abscess
	(ii) infections, such as meningitis, encephalitis, and intracranial abscess
	(iii) metabolic encephalopathy, such as hypoglycemia, electrolyte imbalance, anoxia, hypercarbia, and uremia
	(iv) hyperammonemia from other causes, such as secondary to ureterosigmoidostomy and inherited urea cycle disorders
	(v) toxic encephalopathy from alcohol intake, such as acute intoxication, alcohol withdrawal, and Wernicke encephalopathy
	(vi) toxic encephalopathy from drugs, such as sedative hypnotics, antidepressants, antipsychotic agents, and salicylates
	(vii) organic brain syndrome
	(viii) postseizure encephalopathy

Hepatocellular carcinoma	Verified through medical record review and/or cancer registries

**Table:** (a) End-stage renal disease

Characteristics^a^	Total		Screened positive		Screened negative		Verified ESRD		Randomly selected subcohort^b^	
	*n* = 43 433		*n* = 822		*n* = 42 611		*n* = 620		*n* = 2415	
Demographics										
Median age at enrollment (years, IQR)	39	(33, 46)	42	(33, 49)	39	(33, 46)	41	(35, 48)	39	(33, 45)
Male sex (*n*, %)	34 611	80%	567	69%	34 044	80%	435	70%	1861	77%
Race and ethnicity (*n*, %)										
Non-Hispanic Black	14 720	34%	652	79%	14 068	33%	519	84%	996	41%
Non-Hispanic White	18 068	42%	93	11%	17 975	42%	51	8%	963	40%
Hispanic	5252	12%	55	7%	5197	12%	34	5%	183	8%
Other/unknown	5393	12%	22	3%	5371	13%	16	3%	276	11%
HIV transmission risk (*n*, %)^c^										
Men who have sex with men	20 006	46%	201	24%	19 805	46%	156	25%	1078	45%
Injection drug use	6278	14%	188	23%	6090	14%	142	23%	410	17%
Heterosexual contact	10 576	24%	337	41%	10 239	24%	250	40%	715	30%
Other/unknown	6573	15%	96	12%	6477	15%	72	12%	215	9%
Hepatitis B/C coinfection										
Hepatitis C infection (*n*, %)	8222	19%	313	38%	7909	19%	238	38%	576	24%
Hepatitis B infection (*n*, %)	3938	9%	96	12%	3842	9%	73	12%	234	10%

**Table:** (b) End-stage liver disease

Characteristics^a^	Total		Screened positive		Screened negative		Verified ESLD		Randomly selected subcohort^b^	
	*n* = 41 463		*n* = 2024		*n* = 39 439		*n* = 645		*n* = 2422	

Demographics										
Median age at enrollment (years, IQR)	39	(33, 46)	41	(36, 48)	39	(33, 45)	43	(36, 50)	39	(33, 45)
Male sex (*n*, %)	33 024	80%	1596	79%	31 428	80%	548	85%	1880	78%
Race and ethnicity (*n*, %)										
Non-Hispanic black	14 107	34%	764	38%	13 343	34%	180	28%	992	41%
Non-Hispanic white	17 216	42%	901	45%	16 315	41%	323	50%	985	41%
Hispanic	4803	12%	207	10%	4596	12%	75	12%	186	8%
Other/unknown	5337	13%	152	8%	5185	13%	66	10%	277	11%
HIVtransmissionrisk (*n*, %)^c^										
Men who have sex with men	20 006	48%	772	38%	19 234	49%	262	41%	1,087	45%
Injection drug use	6087	15%	581	29%	5506	14%	193	30%	411	17%
Heterosexual contact	10 576	26%	503	25%	10 073	26%	123	19%	716	30%
Other/unknown	4794	12%	168	8%	4626	12%	66	10%	226	9%
Hepatitis B/C coinfection										
Hepatitis C infection (*n*, %)	8392	20%	1035	51%	7357	19%	375	58%	588	24%
Hepatitis B infection (*n*, %)	3938	9%	345	17%	3593	9%	151	23%	234	10%

^a^Characteristics were measured at enrollment into the cohort with the exception of hepatitis C infection; evidence of hepatitis C infection at enrollment or under observation classified an individual as having infection. Hepatitis B infection was defined by a positive hepatitis B surface antigen or detectable hepatitis B DNA result. Hepatitis C infection was defined as a positive hepatitis C antibody or detectable hepatitis C RNA or genotype result.

^b^The subcohort is the group of randomly selected individuals from contributing cohorts, all of whom underwent comprehensive medical record review.

^c^Men who have sex with men who also reported injection drug use were classified as injection drug use risk.

**Table 3:** Sensitivity, specificity, positive predictive value (PPV), and negative predictive value (NPV) of screening algorithms for end-stage renal disease (ESRD) and end-stage liver disease (ESLD) outcomes among participants in the randomly selected subcohort (*n* = 2,415 for ESRD and *n* = 2,422 for ESLD).

Outcome	Screened positive (*n*)	Verified case (*n*)	Sensitivity	Specificity	PPV	NPV
*End-stage renal disease *						
Overall (diagnosis OR laboratory)	76	62	100%	99%	82%	100%
Diagnosis criteria only	21	17	27%	100%	81%	98%
Laboratory criteria only	58	48	77%	100%	83%	99%
Diagnosis AND laboratory criteria	3	3	5%	100%	100%	98%
*End-stage liver disease *						
Overall (diagnosis OR laboratory)	154	41	100%	95%	27%	100%
Diagnosis criteria only	135	39	95%	96%	29%	100%
Laboratory criteria only	36	8	20%	99%	22%	99%
Diagnosis AND laboratory criteria	17	6	15%	100%	35%	99%
Diagnosis of ascites, SBP, or variceal hemorrhage^a^	62	24	59%	98%	39%	99%

^a^Subgroup of diagnoses used to screen for ESLD.

**Table 4:** Diagnoses and procedure codes for ascertainment of ESRD among NA-ACCORD participants.

End-stage renal disease	
ICD-9-CM codes	Description
581–581.9	Nephrotic syndrome
582–582.9	Chronic glomerulonephritis
583–583.9	Nephritis and nephropathy, not specified as acute or chronic
585–585.9	Chronic kidney disease (CKD)
586	Renal failure, unspecified
588–588.9	Disorders resulting from impaired renal function
593.71–593.73	Vesicoureteral reflux with reflux nephropathy
593.9	Unspecified disorder for kidney and ureter
585.6	End stage renal disease
792.5	Cloudy (hemodialysis) (peritoneal) dialysis effluent
V42.0	Organ or tissue replaced by transplant kidney
V45.1	Renal dialysis status
V56	Encounter for dialysis and dialysis catheter care
V56.0	Extracorporeal dialysis
V56.1	Fitting and adjustment of extracorporeal dialysis catheter
V56.2	Fitting and adjustment of peritoneal dialysis catheter
V56.3	Encounter for adequacy testing for dialysis
V56.31	Encounter for adequacy testing for hemodialysis
V56.32	Encounter for adequacy testing for peritoneal dialysis
V56.8	Other dialysis

**Table 5:** Diagnoses and procedure codes for ascertainment of ESLD among NA-ACCORD participants.

End-stage liver disease	
ICD-9-CM codes	Description
789.5	Ascites
456.0–456.21	Esophageal varices
567.0–567.9	Peritonitis in infectious diseases classified elsewhere
070.0	Viral hepatitis A with hepatic coma
070.4–070.49	Other specified viral hepatitis with hepatic coma
070.6	Unspecified viral hepatitis with hepatic coma
570	Acute and subacute necrosis of liver
571	Chronic liver disease and cirrhosis
571.5	Cirrhosis of liver without mention of alcohol
571.8	Other chronic nonalcoholic liver diseases
572.2	Hepatic encephalopathy
572.3	Portal hypertension
572.4	Hepatorenal syndrome
782.4	Jaundice, unspecified, not of newborn
V42.7	Organ or tissue replaced by transplant liver
54.91	Percutaneous abdominal drainage
39.1	Intra-abdominal venous shunt

## Appendix

See Tables 4 and 5.

## Abbreviations

AASLD: American Association for the Study of Liver Disease
ACASI: Audio computer-assisted self-interviewing
AIDS: Acquired immunodeficiency syndrome
AKI: Acute kidney injury
ALT: Alanine aminotransferase
APRI: Aspartate aminotransferase/platelet ratio index
ART: Antiretroviral therapy
AST: Aspartate aminotransferase
AV: Arteriovenous
DMC: Data Management Core
EASL: European Association for the Study of the Liver
EGD: Esophagogastroduodenoscopy
eGFR: Estimated glomerular filtration rate
ESLD: End-stage liver disease
ESRD: End-stage renal disease
FIB-4: Fibrosis-4
HCC: Hepatocellular carcinoma
HCV: Hepatitis C virus
HD: Hemodialysis
HE: Hepatic encephalopathy
HIV: Human immunodeficiency virus
HIVAN: HIV-associated nephropathy
HMO: Health Maintenance Organization
ICD-9-CM: International Classification of Diseases, 9th Revision, Clinical Modification
IeDEA: International Epidemiologic Databases to Evaluate AIDS
INR: International normalized ratio
MI: Myocardial infarction
NA-ACCORD: North American AIDS Cohort Collaboration on Research and Design
NPV: Negative predictive value
PD: Peritoneal dialysis
PPV: Positive predictive value
PRO: Patient-reported outcome
RRT: Renal replacement therapy
SBP: Spontaneous bacterial peritonitis
SSO: Single sign-on
TIPS: Transjugular intrahepatic portosystemic shunt
US: United States
VACS: Veterans Aging Cohort Study.
